# An Integrated Model of Biphasic Apoptosis in Avian Coccidiosis: Molecular Networks and Host–Parasite Interplay

**DOI:** 10.3390/ani15243528

**Published:** 2025-12-07

**Authors:** Jin Huang, Kang Cheng, Jinrong Wang

**Affiliations:** School of Bioengineering, Henan University of Technology, Zhengzhou 450001, China; huangjin@haut.edu.cn (J.H.); chengkang68@126.com (K.C.)

**Keywords:** avian coccidiosis, apoptosis, host–parasite interplay, IECs, signaling pathway

## Abstract

Coccidiosis is one of the most serious parasitic diseases in poultry, with *Eimeria*-induced apoptosis of IECs recognized as a key pathogenic mechanism. This review systematically delineates the molecular mechanisms governing this apoptotic process. The invasion process of *Eimeria* app. is mediated by the AMA1-RON2 moving junction complex and secreted effector proteins. An integrated model of apoptotic regulation is proposed. This model comprises the mitochondrial, death receptor, and endoplasmic reticulum stress pathways, which are coordinated by signaling hubs, such as PI3K/Akt, NF-κB, and JNK/p38 MAPK, and is further finely modulated by non-coding RNA networks. It is notable that the apoptosis during coccidial infection exhibits a biphasic pattern, where early inhibition supports parasite development and late activation facilitates parasite release and dissemination. Although potential therapeutic targets have emerged for these signaling pathways, how the host precisely switches between different apoptotic pathways remains a current core knowledge gap. Future research needs to thoroughly analyze the molecular logic of host–parasite interaction and ultimately lay a theoretical foundation for developing new strategies targeting the process of cell apoptosis for coccidiosis prevention and control.

## Simple Summary

Coccidiosis is a major disease in chickens. This parasite is very harmful because it intentionally controls a process of cell death in the chicken’s gut. Our review explains how this works. The parasite first gets into cells using a special lock-and-key method. It sends signals to manipulate the cell. We describe how it uses three main pathways to control apoptosis of IECs. It cleverly follows a two-stage pattern. At first, it stops cells from dying, so it can grow safely. Later, it triggers cell death to spread to new cells. Scientists see these signaling pathways as potential targets for new drugs. Understanding these interactions will help create new ways to fight coccidiosis, which is crucial for healthy chickens and stable food production.

## 1. Introduction

Avian coccidiosis is caused by protozoans of the genus *Eimeria*. The disease occurs worldwide and causes ongoing financial losses in poultry farming [1]. *Eimeria* spp. predominantly parasitize the intestinal epithelial cells (IECs) of chickens, where they undergo an obligate intracellular life cycle involving schizogony and gametogony [2]. This replicative cycle drives massive coccidial multiplication and subsequent intestinal damage from large-scale epithelial cell loss. At the peak of infection, pathological changes are especially pronounced, which are characterized by extensive epithelial cell shedding, exposure of the lamina propria, and widening of intercellular spaces. Host cell primary metabolism, including glycolysis, the tricarboxylic acid (TCA) cycle, as well as amino acid biosynthesis, nucleotide synthesis, and lipid metabolism, undergoes significant alterations [3]. These changes impair nutrient absorption and result in growth retardation and reduced feed conversion efficiency [4,5]. Moreover, infected birds are often accompanied by gut microbiota dysbiosis, letting harmful germs colonization and raising death rates in flocks [6].

Most damage to the intestinal mucosa results from apoptosis of the infected IECs [7]. Histopathological observations show that, during late *Eimeria tenella* (*E. tenella*) infection, merozoites proliferate and release extensively. This leads to severe necrosis and sloughing of the cecal epithelium, with apoptosis serving as a principal mechanism driving this extensive cellular loss [8]. Advances in high-throughput sequencing, particularly transcriptomics (RNA-Seq) and proteomics, enable the systematic study of infection-driven changes to host gene expression [9]. By comparing gene expression between infected and uninfected hosts, or hosts infected with strains of different virulence, research has identified thousands of differentially expressed genes (DEGs) [10]. Functional enrichment analyses show these genes are involved in diverse biological processes. These include immune responses, inflammatory reactions, cellular metabolism, cell cycle regulation, and apoptosis [11].

Emerging evidence indicates that coccidian-induced apoptosis is a multilayered network of interconnected signaling pathways. The network is initiated by primary triggers such as direct pathogen invasion and host immune stress, which activate core apoptotic routes-including the mitochondrial and death receptor pathways, endoplasmic reticulum (ER) stress response, and non-canonical mechanisms such as epigenetic and metabolic stress. These pathways ultimately converge on proteolytic cascades mediated by executioner caspases, determining the fate of infected cells. Although omics studies have outlined this framework, details on interpathway crosstalk, temporal dynamics, and stage-specific regulation remain limited.

This review will systematically delineate the molecular network integrating the death receptor, mitochondrial, and ER stress pathways, and discuss the fine-tuning roles of non-coding RNAs. For conceptual clarity, we define the infection timeline as comprising an early phase (approximately 0–24 h post-infection, hpi), focused on parasite establishment and survival, followed by a late phase (approximately 24–120 hpi), characterized by parasite replication and egress. The proposed biphasic apoptosis model is framed within this temporal context. This synthesis aims to clarify *Eimeria* pathogenesis and ultimately support the development of novel interventions against poultry coccidiosis.

## 2. Invasion by *Eimeria*

The invasion of chicken IECs by *Eimeria* species represents a multi-stage process precisely regulated by both host and parasite factors. This complex and evolutionarily conserved mechanism involves a series of molecular interactions and pathological alterations within host cells. Genomic analyses show that about 76% of predicted genes (6700) are expressed across four developmental stages of *E. tenella* (unsporulated oocysts, sporulated oocysts, sporozoites, and merozoites) [12]. This suggests the parasite may express nearly 9000 proteins in its life cycle. Proteomic analyses of *Eimeria necatrix* found 118 proteins directly involved in host cell invasion [13]. Apicomplexan sporozoites share a conserved apical complex that helps them enter host cells [14]. Besides canonical apical organelle proteins like microneme, rhoptry, and dense granule proteins, *Eimeria* invasion also uses noncanonical apical secretory proteins. These include Eimepsin, surface antigen SO7 (SO7), transhydrogenase, lactate dehydrogenase (LDH), and enolase. A key function of many secreted effector proteins from these organelles is to modulate host cell apoptosis in a stage-specific manner, as summarized in Table 1.

Upon ingestion, sporozoites are released from sporulated oocysts by the digestive process in chickens. Sporozoites use gliding motility, powered by a Ca^2+^-dependent actomyosin motor, to move along the intestinal lumen and perform chemotaxis toward the villus tips [15,16]. They first attach to host epithelial cells through GPI-anchored surface antigens (SAG1/SAG2/SAG3). These recognize sulfated lactosylceramides and N-acetylglucosamine/galactose residues on the host brush border via their carbohydrate recognition domains (CRDs). Upon contact, the apical complex is activated [7], leading to secretion of adhesive proteins like thrombospondin-related anonymous protein (TRAP) and micronemal (MIC) complexes [17]. TRAP, through its TSP domain, binds host cell surface integrins (αvβ3/β1) for anchoring, while MIC proteins cluster host membrane lipid rafts to stabilize adhesion and support invasion.

At the onset of invasion, micronemes release apical membrane antigen 1 (AMA1) to the parasite surface. This is followed by secretion of rhoptry neck proteins (RONs), including RON2, RON4, RON5, and RON8 [18]. The transmembrane domain of RON2 inserts into the host cell membrane. Its extracellular D3 loop binds with high affinity to the hydrophobic groove of AMA1, forming the AMA1-RON2 complex. This complex constitutes the core of the moving junction (MJ) [19]. RON4 and RON5 anchor the complex on the host cytoplasmic side [18]. This MJ-dependent invasion mechanism is highly conserved among apicomplexan parasites [20].

Immunization with a sporozoite-specific recombinant EtAMA1 vaccine can elicit anti-AMA1 antibodies that neutralize invasion and confer partial protection against homologous *E. tenella* challenge [21]. In addition, a functional AMA2-RON5 complex has been implicated in MJ assembly [22]. However, AMA2 is not an essential MJ component, and its precise role remains unclear. Direct biochemical or structural evidence for AMA2-RON5 interaction comparable to AMA1-RON2 complex is still lacking. RON3 is required for the correct assembly of the RON2/4/5 complex, and its loss leads to a loosened MJ.

Furthermore, the *E. tenella* serine protease inhibitor 1 (EtSERPIN1) interacts with annexin A2 (ANXA2), promoting lipid-raft aggregation and creating favorable conditions for subsequent invasion steps, including MJ formation [23]. ANXA2 itself functions as an interaction receptor for EtRON2 and serves as a key node in MJ assembly. Antibody blockade of the EtRON2-ANXA2 interaction or interference with EtSERPIN1 markedly reduces sporozoite invasion efficiency [24,25]. By facilitating successful host cell entry, EtSERPIN1 thereby provides an indirect foundation for the early establishment of the parasite, which constitutes a cellular prerequisite for the subsequent phase of apoptosis inhibition. As an interaction hub, ANXA2 represents a potential target for coccidiosis control. However, potential synergistic or competitive effects among ANXA2, EtSERPIN1, and EtRON2 remain to be clarified.

The sporozoite relies on an actin-myosin motor to invade through the MJ and completes invasion within a parasitophorous vacuole (PV) generated by invagination of the host cell membrane [16]. Following invasion, the rhoptry bulbs and associated organelles discharge immunomodulatory effector enzymes, such as the rhoptry proteins ROP18 and ROP16, which phosphorylate host immunity-related GTPases (IRGs) or STAT3. These modifications suppress immune and oxidative stress responses, thereby modulating host immune signaling [26]. Subsequently, dense-granule (GRA) proteins reinforce the structure of the PV and its nutrient exchange channels, consolidating the intracellular parasitic niche. Once internalized, sporozoites differentiate into trophozoites and initiate asexual reproduction (merogony) to produce meronts. Mature meronts rupture to release numerous merozoites, which then invade adjacent epithelial cells, leading to villous atrophy, hemorrhage, and extensive cellular destruction [16,27]. Ultimately, unsporulated oocysts are excreted with the feces, completing the invasion–replication–excretion cycle.

**Table 1:** *Eimeria*-Derived effector proteins and their roles in regulating host cell apoptosis.

Effector Protein	Proposed Mechanism of Action	Effect on Apoptosis (Evidence Level)	Proposed Role in Biphasic Apoptosis	Key References
EtMIC3 (*E. acervulina*)	Binds host CBL protein to inhibit apoptotic signaling.	Inhibition (Exp. confirmed)	Early: Secures the initial intracellular niche.	[7]
EtMIC4 (*E. tenella*)	Activates host EGFR, triggering downstream PI3K/Akt and ERK survival pathways.	Inhibition (Exp. confirmed)	Early: Maintains a viable cellular environment for development.	[28]
EtROP1 (*E. tenella*)	As a rhoptry kinase, inhibits apoptosis by phosphorylating p53 via a kinase-independent mechanism.	Inhibition (Exp. confirmed)	Early: Promotes parasite survival.	[29]
EtROP38 (*E. tenella*)	As a rhoptry kinase, directly suppresses the pro-apoptotic p38 MAPK pathway.	Inhibition (Exp. confirmed)	Early: Counteracts host defense.	[30]
EtROP2 (*E. tenella*)	Activates p38 MAPK signaling, accelerating early schizont development.	Not directly demonstrated (Proposed)	Late: May create a cellular context conducive to egress.	[31]
EtSERPIN1 (*E. tenella*)	Interacts with host ANXA2 to promote lipid-raft aggregation and moving junction formation.	Indirect support (Proposed)	Early: Establishes the infection by facilitating invasion.	[23,25]
EtHGRA9 (*E. tenella*)	As a dense granule protein, influences ER processing, potentially inducing ER stress.	Potential promotion (Proposed)	Phase Transition: Primes the cell for the apoptotic switch. Phase: Promotion? (Potential role)	[11,32]
AMA1-RON2 Complex (*E. tenella*)	Forms the core of the moving junction, essential for invasion.	Prerequisite for infection (Exp. confirmed)	Foundation for both phases: Enables host cell entry.	[18,19,24,25]

Notes: The “Effect on Apoptosis” column indicates the net outcome, with evidence level (Experimentally confirmed or Proposed) in parentheses to distinguish validated chicken-*Eimeria* mechanisms from hypotheses. The “Proposed Role in Biphasic Apoptosis” column frames these effects within the conceptual infection model, defining each protein’s strategic function during early or late phases. For proteins without a direct apoptotic effect (e.g., EtSERPIN1), the role describes how their primary function indirectly supports that phase’s apoptotic profile.

## 3. Dual Roles of *Eimeria* Infection in Chicken Intestinal Cell Apoptosis

### 3.1. A Stage-Dependent Balance Between Survival and Death

Host IECs serve as the primary targets throughout the *Eimeria* life cycle. The infection process follows a distinct temporal framework. The early infection phase (approximately 0–24 hpi) is characterized by cellular invasion and niche establishment, a period during which apoptosis is actively suppressed to ensure parasite survival. Conversely, the late infection phase (approximately 24–120 hpi) is driven by schizogony and merozoite dissemination, processes where the activation of apoptosis becomes prominent.

Consequently, the invasion of these cells initiates a complex “cellular warfare” between host and parasite, in which apoptosis plays a central yet paradoxical role [33]. While mechanical damage and the release of parasite metabolites can directly trigger host apoptotic programs, parasites actively modulate host signaling to counteract these pathways. For instance, sporozoite invasion is accompanied by extensive reorganization of the host cytoskeleton, including the phosphorylation and aggregation of vimentin, which facilitates parasite entry and intracellular parasitism [34]. Mechanistically, disrupting the interaction between EtRON2 and annexin A2 via antibody-mediated blockade significantly reduces host F-actin aggregation, thereby decreasing sporozoite invasion efficiency [25].

At the transcriptomic level, single-cell RNA sequencing (scRNA-seq) reveals profound alterations in intestinal cell composition and states following coccidial infection. These changes are characterized by a substantial loss of *APOB*-expressing enterocytes and a concurrent expansion of proliferating T cells. Notably, infected cells transition toward death-associated states, exhibiting a significant reduction in mitochondrial and cytoplasmic protective functions [35].

### 3.2. Molecular Switches in the Biphasic Apoptosis Model

**Figure 1:**
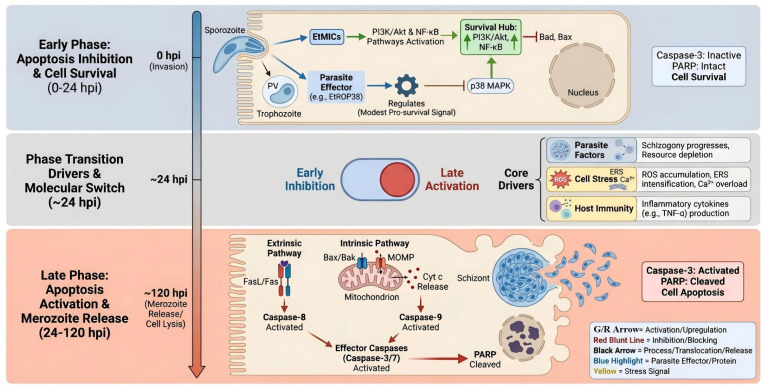
A biphasic model of apoptosis regulation in *E. tenella*-infected chicken IECs. The upper panel (blue background) depicts early infection (0–24 hpi), where parasite effectors suppress apoptosis via PI3K/Akt activation and p38 MAPK inhibition. The lower panel (red background) depicts late infection (24–120 hpi), where accumulated cellular stress and death receptor signaling converge to trigger mitochondrial apoptosis and cell death, enabling parasite release. A central switch symbolizes the transition. G/R arrow, Green and red arrow, The green arrow marks the early stage, while the red arrow marks the late stage.

The transition from the inhibition of early apoptosis to the activation of late-stage apoptosis represents a pivotal event in host-*Eimeria* interactions. Current evidence supports a biphasic pattern, as illustrated in Figure 1, where *E. tenella* initially suppresses host–cell death to secure an intracellular niche but later promotes apoptosis to facilitate egress. During the early intracellular stages, the parasite secretes anti-apoptotic effectors, such as EtMIC4 [28] and EtROP38 [30], which modulate host signaling pathways like EGFR-Akt/ERK and p38 MAPK. Additionally, EtROP1 inhibits host cell apoptosis by binding to and phosphorylating p53 at Thr387, leading to p53 stabilization, notably, this anti-apoptotic effect is independent of its kinase activity [29]. However, as schizogony advances, the signaling landscape shifts towards execution. This transition is characterized by a change in death-receptor adaptor usage: signaling progresses from TNF receptor-associated death domain protein (TRADD) and RIP1-dependent pro-survival mechanisms in the early phase to TRADD and Fas-associated death domain protein (FADD) caspase-8-mediated execution in the mid-to-late phase, thereby engaging the extrinsic apoptotic pathway alongside mitochondrial permeability transition.

This molecular switch is driven by the convergence of parasite developmental programming, cumulative host–cell stress, and mounting immune pressure. Sustained intracellular replication induces mitochondrial dysfunction, Ca^2+^ perturbation, and oxidative damage via reactive oxygen species (ROS) [36], as well as unresolved ER stress. These stressors likely shift the signaling balance toward apoptosis via mediators such as C/EBP homologous protein (CHOP) and c-Jun N-terminal kinase (JNK) [37,38]. Concurrently, host immune responses exacerbate this stress through the production of cytokines and cytotoxic effectors like TNF-αand FasL [39,40], which overwhelm early NF-κB mediated survival signals. Furthermore, transcriptomic data suggest that a network of non-coding RNAs dynamically modulates these pathways. Ultimately, a critical threshold is reached where parasite-derived cues, cellular damage, and host death signals collectively trigger the late apoptotic wave.

## 4. Execution Platform of *Eimeria*-Induced Apoptosis in Chicken Intestinal Cells

In the context of *Eimeria* infection, multiple canonical and non-canonical signaling pathways are activated or inhibited, collectively determining the ultimate fate of IECs.

### 4.1. The Central Role of the Mitochondrial Pathway

In the regulation of chicken IECs apoptosis triggered by coccidial infection, the mitochondria-mediated intrinsic pathway plays a pivotal role [41]. Within 2–6 h post sporozoite invasion, intracellular free Ca^2+^ concentrations increase significantly [42]. This rise activates calcium-dependent proteases, such as calpain. This also disrupts mitochondrial membrane stability. Calcium overload directly induces the opening of the mitochondrial permeability transition pore (mPTP) [36,43]. A burst of mitochondrial ROS and nitric oxide (NO) synergizes with elevated Ca^2+^ signaling. This leads to oxidative injury characterized by elevated malondialdehyde (MDA) levels, imbalance in the activities of the antioxidant enzymes superoxide dismutase (SOD) and catalase (CAT), and lipid peroxidation [36]. At 18–24 h post-infection (hpi), the pro-apoptotic protein Bcl-2-associated X protein (Bax) is activated and translocates from the cytoplasm to mitochondria. The anti-apoptotic protein B-cell lymphoma 2 (Bcl-2) is downregulated, resulting in a markedly elevated Bax/Bcl-2 ratio [44]. Bax oligomerization induces conformational changes in voltage-dependent anion channel (VDAC)/adenine nucleotide translocator (ANT) complexes and promotes persistent mPTP opening. It also causes a pronounced decrease in mitochondrial membrane potential (MMP or ΔΨm), leading to mitochondrial outer membrane permeabilization (MOMP), which is a distinct process from mPTP opening. While sustained mPTP opening (an inner membrane event) can cause swelling and secondary rupture of the outer membrane, MOMP is directly mediated by Bax/Bak oligomerization and can occur independently. Thus, the activation and oligomerization of Bax represent key upstream events that initiate the apoptotic cascade.

However, the mechanisms underlying Bax activation and the loss of mitochondrial membrane potential during *Eimeria*-induced apoptosis have not been fully understood. In mammalian models, Bax activation may occur through the stress-induced BH3-only proteins. Whether a similar mechanism operates in *Eimeria*-infected chicken cells is an open question that requires direct demonstration. These proteins may directly bind and trigger Bax oligomerization. Loss of MMP may result from ion leakage through Bax/Bak pores. It may also occur indirectly via alterations in associated channels such as VDAC/ANT-mediated mPTP. Enhanced MOMP facilitates the release of cytochrome c (cyt c), Smac/DIABLO, endonuclease G (Endo G), and apoptosis-inducing factor (AIF) from mitochondria into the cytoplasm. This release initiates the cyt c-dependent apoptotic cascade. Nevertheless, the regulatory mechanisms controlling cyt c release remain controversial [1]. Furthermore, the possibility that *Eimeria* species may directly modulate these processes with unique parasite-derived effector proteins or unknown pore-forming mechanisms cannot be excluded. Between 24 and 72 hpi, cytosolic cyt c binds Apaf-1 to form the apoptosome [45]. This complex activates caspase-9, which subsequently cleaves procaspase-3 and procaspase-7 into their active forms [46]. Activated caspase-3 targets poly(ADP-ribose) polymerase (PARP) and the inhibitor of caspase-activated DNase (ICAD). This action leads to CAD-mediated nuclear DNA fragmentation with characteristic 200 bp laddering. Ultimately, these events result in programmed death of IECs [47,48].

Pharmacological evidence supports the central role of mitochondria in apoptosis. Treatment with the mPTP inhibitor cyclosporin A (CsA), the cyt c reductant TMPD+Asc, or the caspase-9 inhibitor Z-LEHD-FMK significantly suppresses apoptosis in *E. tenella* host cells [49,50]. Although mitochondrial apoptosis is primarily executed through caspase-mediated proteolytic cascades, studies in human mesothelioma cell lines have shown that broad-spectrum caspase inhibitors (e.g., Z-VAD-FMK) cannot completely prevent DNA damage, implying the existence of non-caspase-dependent apoptotic pathways [51]. Similarly, research in primary rat proximal tubule (rPT) cells demonstrated concurrent activation of caspase-dependent (cyt c-caspase) and caspase-independent (BNIP3/AIF/Endo G) pathways [52].

Endo G and AIF are released from mitochondria and translocate to the nucleus via nuclear localization signals (NLS) and PARP-1-dependent mechanisms, respectively. There, they induce single-strand DNA breaks and fragmentation independent of caspase activation [53]. Even without caspase activity, mitochondrial release of AIF and Endo G can drive cells toward irreversible death. This alternative apoptotic route has been extensively studied in cancer, neurodegenerative disorders, and viral infections [54,55]. Although caspase-independent pathways have received limited attention in coccidian-induced apoptosis, experimental evidence shows that during *E. tenella* infection (24–120 hpi), cytosolic levels of Endo G and AIF increase significantly. mPTP opening coordinates their release and activates a mitochondrial caspase-independent apoptotic pathway, a process effectively blocked by CsA [8]. These findings confirm a caspase-independent mitochondrial death pathway in *E. tenella*-induced apoptosis.

The mitochondrial pathway is the most thoroughly characterized apoptotic mechanism and serves as the central execution route of coccidian-induced apoptosis. Nonetheless, key upstream events remain elusive, such as the precise molecular mechanisms of Bax activation. While extrapolations from mammalian systems are plausible, *Eimeria* species may directly modulate these processes with unique parasite-derived effector proteins. The ongoing debate regarding the mechanism of cyt c release also suggests that unknown, parasite-specific membrane pore-forming mechanisms may exist. Therefore, investigating MOMP induced by coccidial infection can validate classic apoptotic pathways and may reveal unique strategies by which apicomplexan parasites manipulate host cell fate.

### 4.2. Death Receptor Pathway

Activation of the extrinsic death receptor pathway depends on engagement of cell-surface death receptors (DRs) by their cognate ligands. Canonical death receptors include Fas (CD95/APO-1), TNFR1, and TRAIL receptors (DR4/DR5). These bind to FasL, TNF-α, and TRAIL, respectively [56]. Ligand binding induces receptor trimerization and conformational changes within intracellular death domains (DDs). Trimerized receptors signal through homotypic DD-DD interactions with adaptor proteins, principally TRADD and FADD. Fas and TRAIL receptors directly recruit FADD, whose death effector domain (DED) subsequently recruits DED-containing procaspase-8/10, thereby initiating the extrinsic apoptotic cascade. Together, the receptor, FADD, and procaspase-8/10 assemble the death-inducing signaling complex (DISC) [57].

Upon TNF binding to TNFR1, TRADD is recruited to the receptor complex, where it facilitates recruitment of additional signaling molecules, including receptor-interacting protein kinase 1 (RIPK1) and TRAF2. Under certain conditions, it also facilitates the recruitment of the death adaptor FADD. FADD subsequently recruits procaspase-8 (and in some species procaspase-10), forming a cytosolic death-signaling platform termed complex II [58]. Within this complex, procaspase-8 molecules undergo proximity-induced dimerization and autoproteolytic processing, resulting in the generation of active caspase-8 [54]. Active caspase-8 then cleaves downstream effector caspases (primarily caspase-3, -6, and -7), which degrade critical intracellular substrates including nuclear lamins and DNA repair enzymes, producing characteristic apoptotic features such as chromatin condensation, DNA fragmentation, and apoptotic body formation.

Importantly, when caspase-8 activity is inhibited or absent, TNFR1 signaling shifts from apoptosis toward necroptosis through the interaction of RIPK1/RIPK3, leading to necrosome formation and ultimately triggering MLKL-mediated necroptotic death [59]. The mechanisms by which *Eimeria* infection modulates the balance among these TNFR1-associated outcomes remain poorly understood, representing a critical knowledge gap.

Investigations of *E. tenella* have demonstrated that the death receptor pathway plays a pivotal role in parasite-induced host cell apoptosis [60]. Expression levels of TRADD and FADD correlate significantly with host cell apoptotic rates, indicating their central involvement in this process [39]. Further investigations revealed that TRADD and FADD exhibit opposing functions at different stages of infection. During early infection (4 hpi), TRADD suppresses apoptosis by downregulating RIP1, thereby favoring parasite survival. Conversely, during mid-late stages (24–120 hpi), the TRADD-FADD-caspase-8 axis promotes apoptosis [61]. This temporal switch suggests that *E. tenella* strategically modulates host apoptosis to meet stage-specific developmental requirements, initially preserving the intracellular niche, then facilitating egress and dissemination. Supporting this model, FasL expression increases significantly in cecal tissues at 10 days post-infection [40], confirming activation of the extrinsic pathway during coccidiosis.

The opposing roles of TRADD and FADD at different infection stages reveal a sophisticated spatiotemporal regulatory strategy employed by *Eimeria*. This regulatory plasticity indicates that apoptosis functions not merely as a virulence factor or host defense, but as a precisely orchestrated biological process. The process is modulated to fulfill stage-specific developmental requirements. Fundamental questions remain: What signals govern this regulatory switch? Are they intrinsic developmental programs of the parasite, or do they originate from dynamic changes within the host microenvironment?

## 5. The Role of ER Stress Pathway in *Eimeria*-Induced Apoptosis

The ER is the central organelle for protein synthesis, folding, and modification. Pathogen infection can disrupt ER homeostasis, triggering the unfolded protein response (UPR). If stress persists or becomes excessive, apoptotic programs are initiated [62]. Apicomplexan parasites such as *Toxoplasma gondii* are known to induce apoptosis in neural stem cells and IECs via ER stress-mediated pathways [32]. For *Eimeria* species, however, direct molecular evidence confirming their ability to induce host-cell apoptosis via canonical UPR-apoptosis pathways has yet to be established, although accumulating data suggest a potential mechanistic link. Time-series transcriptomic analyses show that *E. tenella* infection induces early transcriptional upregulation of UPR-related genes in host cells [11], indicating potential ER stress activation. This has led to the hypothesis that ER stress may serve as an upstream trigger and amplifier for the apoptotic cascade. In support of this notion, the coccidian protein EtHGRA9 has been reported to influence the expression of ER processing proteins [63]. It is therefore plausible that the extensive intracellular replication of coccidia imposes a substantial protein-folding burden on the ER, and that persistent stress may contribute to apoptosis. Moreover, Ca^2+^, a key factor in sporozoite invasion, is regulated not only by extracellular influx and plasma membrane channels but also by ER inositol 1,4,5-trisphosphate (IP_3_) and ryanodine receptors [43], providing another potential link to ER homeostasis.

Despite these indications, the current evidence remains largely correlational rather than causative. Direct experimental demonstration of the activation of key UPR sensors (e.g., PERK phosphorylation, IRE1α-dependent XBP1 mRNA splicing, ATF6 cleavage) and their downstream effectors (e.g., CHOP, GRP78) in *Eimeria*-infected chicken IECs is still lacking. Research on how coccidial infection modulates canonical ER stress pathways, such as the PERK-eIF2α-ATF4-CHOP axis, IRE1-XBP1-JNK axis, and ATF6-mediated ER-associated degradation (ERAD)-apoptosis switching, remains limited. While transcriptomic changes and the reported function of EtHGRA9 provide valuable clues, direct molecular connections to apoptotic endpoints have not been experimentally established. Thus, in the context of *Eimeria* infection, ER stress may not act as an independent trigger of apoptosis, but likely serves as a signal amplifier and integrative hub. This role involves tight coupling with mitochondria through Ca^2+^ cycling and the potential transmission of stress signals into the core apoptotic machinery via pathways, such as CHOP and JNK. To move from correlation to causation, future studies should first quantify relevant markers (e.g., CHOP, p-PERK, XBP1 splicing) and then, using sensor-specific inhibitors, experimentally confirm that UPR pathway activation is necessary for Eimeria-induced apoptosis.

The extrinsic (death receptor-mediated), intrinsic (mitochondrial-mediated), and ER stress-mediated apoptotic pathways do not function in isolation. Instead, they engage in extensive crosstalk through key molecular nodes, forming a highly integrated apoptotic regulatory network. This integration is exemplified by Bid, a pro-apoptotic Bcl-2 family protein and a substrate of caspase-8. Upon activation of the extrinsic pathway, caspase-8 cleaves Bid to generate truncated Bid (tBid) [64]. tBid subsequently translocates to the mitochondria, where its BH3 domain promotes MOMP. This process amplifies death receptor signals and transmits them to the mitochondrial machinery [65]. ER stress contributes to this network primarily through the CHOP and JNK axes. CHOP transcriptionally dysregulates the Bcl-2 family balance by upregulating the pro-apoptotic proteins Bim and Puma while downregulating the anti-apoptotic protein Bcl-2 [37]. Meanwhile, JNK activated by the ER stress sensor IRE1α, phosphorylates to enhance Bim activity, and can also phosphorylate to inhibit Bcl-2 [66]. Together, these actions promote MOMP through both transcriptional and post-translational mechanisms, thereby channeling ER stress signals into the mitochondrial apoptotic cascade. The ultimate convergence of these three pathways results in the activation of effector caspases-3 and -7, which cleave key cellular substrates and reinforce apoptosis through positive feedback amplification, driving the cell into irreversible programmed death.

## 6. Regulatory Pathways of *Eimeria*-Induced Apoptosis in Chicken Intestinal Cells

### 6.1. The Anti-Apoptotic Role of the PI3K/Akt Pathway in Eimeria Infection

The PI3K/Akt pathway is a critical survival signaling axis that regulates cell proliferation, differentiation, apoptosis, and migration [67]. In various pathogen-host interaction models, PI3K/Akt signaling has been identified as a key host pathway manipulated by pathogens to promote their survival. Apicomplexan parasites, including *Eimeria* and *Toxoplasma gondii*, commonly activate this host pathway to suppress apoptosis [66,68]. During early *Eimeria* infection, the parasite transiently inhibits host cell death to secure its intracellular niche, a strategy corroborated by observed PI3K/Akt activation in *E. tenella* infection models [69].

Coccidia are presumed to activate phosphoinositide 3-kinase (PI3K) via secreted effector proteins or the engagement of surface receptors, generating PIP_3_ and leading to the phosphorylation of Akt (protein kinase B). Activated Akt phosphorylates and inactivates multiple downstream pro-apoptotic proteins, including Bad, FOXO, and GSK3β. By simultaneously inhibiting several key apoptotic regulators, *Eimeria* establishes a robust anti-apoptotic network within host cells, thereby promoting intracellular survival. However, direct molecular confirmation of the entire signaling cascade remains limited.

Epidermal growth factor receptor (EGFR) is a typical upstream activator of the PI3K/Akt pathway. *E. tenella* microneme protein 4 (EtMIC4), which contains EGF-like domains, can activate host EGFR signaling, thereby regulating downstream Akt and ERK pathway components, suppressing apoptosis, and enhancing infection efficiency [28]. Treatment of infected cells with PI3K inhibitors such as LY294002 significantly increases early apoptosis rates, confirming the crucial role of PI3K/Akt signaling in early coccidial infection [69]. This pathway exerts its anti-apoptotic effects by phosphorylating Bad, restricting mPTP opening, and inhibiting caspase-9/3 activity. The early activation of PI3K/Akt, as exemplified by EtMIC4’s co-option of upstream EGFR signaling, constitutes a key molecular strategy whereby *Eimeria* hijacks host survival pathways to secure a viable intracellular niche during the initial phase of infection. This mechanism not only identifies potential drug targets but also reveals an invasion strategy centered on mimicking and amplifying endogenous survival cues to achieve silent parasitism. Nonetheless, the complete signaling cascade from EtMIC4 to complete Akt activation remains to be elucidated.

### 6.2. The Dual Role of NF-κB Pathway in Eimeria-Induced Apoptosis

The nuclear factor-κB (NF-κB) signaling pathway orchestrates host inflammatory, immune, and survival responses. Canonically, NF-κB activation maintains cell viability by inducing the transcription of anti-apoptotic genes such as Bcl-2, Bcl-xL, c-FLIP, and inhibitor of apoptosis proteins (IAPs) [70,71]. Under specific conditions, however, NF-κB can also promote apoptosis [72].

During *Eimeria* infection, this pathway exhibits context-dependent duality. On the one hand, infection by several *Eimeria* species, including *E. tenella* and *E. acervulina*, activates NF-κB signaling, inducing release of pro-inflammatory cytokines such as IL-1β, IL-6, and TNF-α, which mediate the intestinal pathology of coccidiosis [73,74]. On the other hand, *E. acervulina* may exploit NF-κB’s anti-apoptotic effects to suppress host cell apoptosis and facilitate intracellular development [7], suggesting species or context-specific regulatory mechanisms. Conversely, inhibition of NF-κB activation has been reported in other studies [75], suggesting divergent regulatory strategies. Collectively, these findings indicate that NF-κB output depends on parasite species, infection stage, and host cell context. Within the biphasic apoptosis model, NF-κB’s role is equally context-dependent: its early, anti-apoptotic function may support parasite establishment, while its sustained activation in the late phase may contribute to the pro-inflammatory and potentially pro-apoptotic microenvironment that facilitates parasite egress and tissue dissemination.

Within this network, the PI3K/Akt pathway acts as a major upstream regulator: Akt phosphorylates IκB kinase (IKK), promoting IκB degradation and thereby releasing NF-κB for nuclear translocation and transcriptional activation [76]. This PI3K/Akt-NF-κB signaling axis likely represents a central mechanism by which *Eimeria* fine-tunes the balance between inflammation and apoptosis inhibition during infection.

The paradoxical roles of NF-κB, as both a driver of inflammation and a supporter of cell survival, underscore the dynamic balance between host defense and parasite immune evasion. Rather than simply activating or suppressing this pathway, *Eimeria* likely exploits its regulatory flexibility to maintain host cell survival while minimizing excessive inflammation. Future studies integrating species-specific and stage-specific analyses are required to develop a unified model of NF-κB modulation during coccidial infection.

### 6.3. The Role of JNK/p38 MAPK Pathways in Eimeria-Induced Apoptosis

MAPK pathways are evolutionarily conserved signaling modules that transduce extracellular stimuli into precise intracellular responses, regulating proliferation, differentiation, and apoptosis. Among them, JNK and p38 MAPK serve as key mediators of stress-induced apoptosis [77]. *Eimeria* infection activates host MAPK pathways [78], and inhibition of MAPK signaling significantly reduces *E. tenella* sporozoite invasion [79], highlighting the importance of these pathways in host–parasite interactions.

Upon activation, JNK and p38 MAPK exert their pro-apoptotic functions primarily through two mechanisms. First, via transcription-dependent regulation, they phosphorylate and activate downstream transcription factors such as c-Jun to modulate the expression of apoptosis-related genes. Second, they directly phosphorylate mitochondrial or cytoplasmic pro-apoptotic proteins, including Bim and BAD, thereby triggering the mitochondrial apoptotic pathway [31]. Interestingly, *Eimeria* has evolved refined mechanisms to counter-regulate these host defenses. *E. tenella* rhoptry protein EtROP38 can enter host cells and specifically suppress p38 MAPK activation, thereby inhibiting apoptosis and favoring parasite survival [30]. In contrast, EtROP2, a distinct member of the ROP kinase family, activates p38 MAPK signaling and is associated with accelerated early schizont [80]. Given the established pro-apoptotic role of p38 MAPK under stress conditions, it is plausible that EtROP2 activity could contribute to apoptosis in the late phase. However, a direct causal relationship between EtROP2 and host cell apoptosis has not yet been established.

These opposing regulatory effects of EtROP38 and EtROP2 on p38 MAPK demonstrate the precision and diversity of coccidian effector protein functions. Rather than operating through simple “on/off” mechanisms, *Eimeria* employs a finely tuned, regulatory strategy, allowing it to differentially modulate host signaling for optimal intracellular adaptation. This temporal regulation of the JNK/p38 MAPK pathways, which early inhibition to ensure survival followed by late activation to facilitate egress, exemplifies the sophisticated implementation of the biphasic apoptosis model by the parasite.

## 7. Fine-Tuning by Noncoding RNAs

Recent studies have identified noncoding RNAs (ncRNAs), particularly microRNAs (miRNAs) and long noncoding RNAs (lncRNAs), as crucial regulators in gene expression networks, playing essential roles in host–pathogen interactions [81,82]. In the context of avian coccidiosis, high-throughput studies have revealed extensive reprogramming of both miRNA and lncRNA expression, providing a rich resource for identifying key regulators of the host response [83,84].

### 7.1. The Role of lncRNAs in the Apoptosis of IECs Induced by Eimeria

LncRNAs regulate gene expression through multiple mechanisms, functioning as molecular decoys, scaffolds, or signaling molecules. In chicken coccidiosis research, lncRNAs have recently emerged as a new focus. Among the lncRNAs dysregulated during *E. tenella* infection, the host lncRNA BTN3A2 emerges as a functionally characterized example. *E. tenella* infection upregulates BTN3A2, which subsequently attenuates excessive inflammatory responses by suppressing NF-κB signaling [85]. Although this study primarily addressed inflammation regulation, given the essential role of NF-κB in cell survival, the BTN3A2-mediated modulation of this pathway represents a compelling, albeit indirect, mechanism through which lncRNAs could influence the apoptotic fate of infected cells.

In other infectious models, certain lncRNAs have been shown to directly mediate apoptosis through the JNK and other signaling pathways [86], providing a conceptual framework for exploring lncRNA-mediated apoptotic regulation in coccidial infection. However, to date, no specific lncRNA has been experimentally verified to directly modulate apoptotic pathways in chicken coccidiosis. The underlying mechanisms remain to be elucidated.

### 7.2. The Role of miRNAs in the Apoptosis of IECs Induced by Eimeria

miRNAs are endogenous, short ncRNAs (approximately 20–22 nucleotides) that act as pivotal post-transcriptional regulators of gene expression [87]. High-throughput sequencing of cecal tissues from *E. tenella*-infected chickens has revealed significant differential expression of miRNAs, particularly those enriched in pathways governing immunity and cell survival [84]. Notably, gga-miR-2954 has been identified as one of the most significantly upregulated miRNAs. While functional studies demonstrated that gga-miR-2954 suppresses inflammatory cytokine production in sporozoite-stimulated cells by directly targeting the RAR-related orphan receptor C (*RORC*) gene [88], its potential role in directly regulating core apoptotic genes remains to be elucidated.

**Figure 2:**
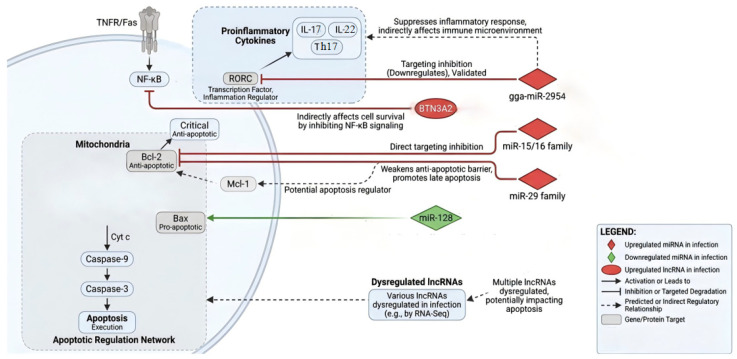
Proposed regulator network of miRNAs and lncRNAs in *Eimeria*-induced apoptosis. The figure illustrates specific miRNAs and lncRNAs with significant expression changes during *Eimeria* infection, and their targeting relationships with core apoptotic and inflammatory pathway genes based on sequence prediction or preliminary validation. Solid lines indicate relationships supported by partial functional evidence, while dashed lines represent predicted relationships based on homology or correlation.

In addition to inflammation-related miRNAs, expression datasets suggest the involvement of miRNAs known to target apoptosis regulators in other species. For instance, the miR-15/16 family and miR-29, which established post-transcriptional repressors of the anti-apoptotic protein Bcl-2, are dysregulated during infection [89,90]. Furthermore, miRNAs with potential targets in the intrinsic pathway, such as miR-128 (a proposed regulator of BAX [91]), also exhibit altered expression. Collectively, these findings suggest a complex miRNA network that may fine-tune the biphasic apoptotic process by simultaneously modulating inflammatory signals and apoptotic executioners. This potential regulatory network is summarized in Figure 2.

However, it is critical to acknowledge that for the majority of these dysregulated miRNAs, direct functional evidence confirming their roles in *Eimeria*-induced apoptosis is currently lacking. Consequently, future research must shift from correlative profiling to mechanistic investigations, utilizing gain- or loss-of-function experiments to verify whether these miRNAs regulate apoptosis by directly targeting the mRNAs of core apoptotic genes.

## 8. Challenges and Perspectives: Limitations of Extrapolating Mammalian Apoptotic Mechanisms to Avian Systems

While this review proposes a model of apoptosis during *Eimeria* infection, with key experimental evidence and proposed mechanisms summarized in Table 2. It is crucial to acknowledge that many underlying mechanisms are currently extrapolated from mammalian systems. Critical molecular events, such as the precise activation cascade of Bax and the functional contribution of UPR sensors to cell death, are well-characterized in mammals but lack experimental verification in the chicken-*Eimeria* context. Furthermore, current evidence linking pathways like ER stress and specific non-coding RNAs to apoptosis is predominantly correlative rather than causative, as it is derived largely from transcriptomic data. Consequently, future research must prioritize functional validation within avian systems. Elucidating the degree of conservation in these apoptotic pathways and uncovering host- or parasite-specific adaptations are essential steps toward developing precise, targeted anti-coccidial interventions.

## 9. Conclusions

The interaction between *Eimeria* and its host, particularly during invasion and the subsequent apoptosis of infected cells, constitutes a complex, multi-level molecular network. Despite this complexity, the network orchestrates a distinct biphasic outcome regarding host cell fate. In the early stages of infection, apoptosis is suppressed through the concerted action of parasite effectors (e.g., EtMIC4 and EtROP38), which activate the PI3K/Akt survival pathway and inhibit pro-apoptotic MAPK signaling. Concurrently, the NF-κB pathway balances the immune response with cell survival, while JNK/p38 MAPK mediates stress signals. Additionally, numerous non-coding RNAs (ncRNAs) are enriched in pathways governing immunity and cell survival. Conversely, the late phase of infection is characterized by the convergence of the mitochondrial pathway (driven by Bax activation and MOMP), the death receptor pathway (via FasL and TRADD/FADD signaling), and the amplification of ER stress signals. This critical transition from survival to death is likely driven by a combination of autocrine/paracrine signals and, crucially, the parasite’s own developmental checkpoint: the completion of schizogony, which shifts the parasitic strategy from intracellular survival to dissemination.

Despite extensive research leading to proposed control measures—such as recombinant vaccines and caspase inhibitors—significant challenges persist. Current mechanistic understanding is largely extrapolated from mammalian apoptosis models or studies of other apicomplexan parasites. Direct experimental evidence confirming that these specific molecular events drive apoptosis during *Eimeria* infection in avian hosts remains limited. Furthermore, the reliance on in vitro cell models in most current studies fails to fully recapitulate the intricate dynamics of host–parasite interactions within the living organism.

To bridge these gaps, future research must prioritize physiologically relevant models, such as intestinal organoids or in vivo gene editing systems (e.g., CRISPR/Cas9). Utilizing tools like stage-specific parasite mutants will be essential to precisely dissect the molecular switch between cell survival and death. Elucidating the specific apoptotic pathways induced by *Eimeria* in chicken IECs will not only advance our understanding of host–parasite co-evolution but also provide novel, precise molecular targets for the development of next-generation anti-coccidial strategies.

The interaction between *Eimeria* and its host, particularly during invasion and the subsequent apoptosis of infected cells, involves a dynamic interplay that forms a complex, multi-level network. However, this network orchestrates a clear biphasic outcome in host cell fate. In the early stages of infection, the suppression of apoptosis is orchestrated through the concerted action of parasite effectors like EtMIC4 and EtROP38, which activate the PI3K/Akt survival pathway and inhibit pro-apoptotic MAPK signaling, respectively. Concurrently, the NF-κB pathway balances immune response with cell death, while JNK/p38 MAPK acts as a stress signal mediator. Numerous ncRNAs are enriched in pathways governing immunity and survival. Conversely, the late phase of infection is executed via the convergence of the mitochondrial pathway (driven by Bax activation and MOMP), the death receptor pathway (via FasL and TRADD/FADD signaling), and the amplification of stress signals from the ER. The switch from the early to the late phase is likely driven by a combination of autocrine/paracrine death signals from the initially infected cells and, crucially, the parasite’s own developmental checkpoint: the completion of schizogony, which shifts its strategy from survival to dissemination.

Although extensive research has been conducted on this network, leading to proposed control measures such as recombinant vaccines and caspase inhibitors to reduce coccidial invasion and host cell apoptosis, key challenges persist. Our understanding of the underlying molecular mechanisms is largely extrapolated from mammalian apoptosis models or studies of other apicomplexan parasites. Direct experimental confirmation that these molecular events occur during *Eimeria* infection is still lacking. Furthermore, most current studies employ in vitro cell models, which fail to fully recapitulate the complex dynamics of host–parasite interactions within a living host.

Future research must employ more physiologically relevant models, such as organoids or in vivo gene editing, and utilize tools like stage-specific parasite mutants to precisely dissect this molecular switch. Deciphering the apoptotic pathway induced by coccidia in chicken intestinal cells will advance our knowledge of the intricate co-evolutionary dynamics between host and parasite and provide novel, precise molecular targets for developing new anti-coccidial strategies.

## Figures and Tables

**Table 2:** Summary of key evidence for apoptotic pathways in chicken *Eimeria* infection.

Pathway	^1^Direct Experimental Evidence	^2^Proposed/Inferred Mechanisms
Mitochondrial	Bax/Bcl-2 ratio ↑; Cyt c release; Caspase-3 activation [44,46]. Inhibition of apoptosis by mPTP blocker (CsA) and caspase-9 inhibitor [49,50]. Caspase-independent apoptosis via AIF/Endo G release [8].	Precise molecular trigger for Bax activation (e.g., specific BH3-only proteins). The exact composition and regulation of the mPTP.
Death Receptor	Temporal, stage-specific roles of TRADD and FADD [39,61]. Upregulation of FasL during late infection [61].	Detailed composition and regulation of the DISC/complex II. Molecular switch mechanism governing the transition from survival to death signaling via TNFR1.
ER Stress	Transcriptomic upregulation of UPR-related genes [11]. Effector protein EtHGRA9 influences ER processing protein expression [63].	Direct evidence for PERK, IRE1, ATF6 sensor activation. Potential association of CHOP or JNK in driving apoptosis.
PI3K/Akt	Activation during early infection; apoptosis increase upon inhibition (e.g., LY294002) [69]. Effector protein EtMIC4 activates EGFR/PI3K/Akt signaling [28].	Complete signaling cascade from other parasite effectors to Akt activation.
NF-κB	Activation and pro-inflammatory cytokine induction [73,74]. Context-dependent anti-apoptotic role [7].	A unified model explaining its context-dependent pro-survival vs. pro-death outcomes.
JNK/p38 MAPK	Effector protein EtROP38 inhibits p38 MAPK to suppress apoptosis [30]. Effector protein EtROP2 activates p38 MAPK [80]. MAPK inhibition reduces sporozoite invasion [80].	Comprehensive mapping of all upstream activators and downstream apoptotic targets.
Non-coding RNAs	Widespread dysregulation of miRNAs and lncRNAs during infection [84,85]. gga-miR-2954 inhibits inflammation by targeting RORC [88].	Direct functional evidence for specific miRNAs/lncRNAs in regulating core apoptotic genes (e.g., Bcl-2, Bax) via gain/loss-of-function experiments.

^1^Direct Experimental Evidence: In Chicken-*Eimeria* Models; ^2^Proposed: Based on analogy or correlation. ↑: Increase.

## Data Availability

No new data were created or analyzed in this study.
